# Identifying essential genes in bacterial metabolic networks with machine learning methods

**DOI:** 10.1186/1752-0509-4-56

**Published:** 2010-05-03

**Authors:** Kitiporn Plaimas, Roland Eils, Rainer König

**Affiliations:** 1 Department of Bioinformatics and Functional Genomics, Institute of Pharmacy and Molecular Biotechnology, Bioquant, University of Heidelberg, Im Neuenheimer Feld 267, 69120 Heidelberg, Germany; 2 B080 Division of Theoretical Bioinformatics, German Cancer Research Center (DKFZ), Im Neuenheimer Feld 280, 69120 Heidelberg, Germany

## Abstract

**Background:**

Identifying essential genes in bacteria supports to identify potential drug targets and an understanding of minimal requirements for a synthetic cell. However, experimentally assaying the essentiality of their coding genes is resource intensive and not feasible for all bacterial organisms, in particular if they are infective.

**Results:**

We developed a machine learning technique to identify essential genes using the experimental data of genome-wide knock-out screens from one bacterial organism to infer essential genes of another related bacterial organism. We used a broad variety of topological features, sequence characteristics and co-expression properties potentially associated with essentiality, such as flux deviations, centrality, codon frequencies of the sequences, co-regulation and phyletic retention. An organism-wise cross-validation on bacterial species yielded reliable results with good accuracies (area under the receiver-operator-curve of 75% - 81%). Finally, it was applied to drug target predictions for *Salmonella typhimurium*. We compared our predictions to the viability of experimental knock-outs of *S. typhimurium *and identified 35 enzymes, which are highly relevant to be considered as potential drug targets. Specifically, we detected promising drug targets in the non-mevalonate pathway.

**Conclusions:**

Using elaborated features characterizing network topology, sequence information and microarray data enables to predict essential genes from a bacterial reference organism to a related query organism without any knowledge about the essentiality of genes of the query organism. In general, such a method is beneficial for inferring drug targets when experimental data about genome-wide knockout screens is not available for the investigated organism.

## Background

By definition, essential proteins of a cellular organism are necessary to live and replicate, and are therefore attractive targets for antimicrobial treatments. However, experimentally assaying the essentiality of their coding genes is error prone when done in high throughput. Additionally, experimental screens are resource intensive and not feasible for all organisms, as typically, for each gene a knock-out strain needs to be constructed. Besides this, pathogenic bacterial organisms are hazardous to cultivate and therefore need higher laboratorial efforts. The metabolism of a cell is substantial for maintaining life and growth, and hence metabolic enzymes have been successfully targeted by antibiotics inhibiting essential processes in bacterial genomes [1].

Several computational techniques have been developed to identify essential genes *in silico*. Flux balance analyses (FBA) is widely used to assess the essentiality of genes [2]. However, FBA approaches need clear definitions of nutrition availability and biomass production under specifically given environmental conditions [3]. Descriptors for enzymes in the metabolic network were put up by graph theoretical approaches and were used to identify drug targets in micro-organisms. Concepts of choke-points and load-points were successfully applied to estimate the essentiality of an enzyme [4-6]. The term 'damage' was used to assess enzymes that may serve as drug targets when their inhibition influences a substantial number of downstream reactions and products [7]. In a previous study, we examined the ability of the network to obtain the products of a knocked-out reaction from its educts via alternative pathways and used this method to detect potential drug targets for *P. falciparum *[6]. Various descriptors for centrality of a node in a network have been successfully applied and supported detecting essential proteins in protein-protein interaction networks [8-12]. It was shown that proteins which have a more central position evolve more slowly and are more likely to be essential for survival [9]. Also sequence features like codon usage, GC-content and localization signals were used for predicting essential genes. They were successfully applied for inferring essential genes from *S. cerevisiae *to the less studied yeast strain *S. mikatae *[13]. Additionally, phyletic retention has been found to be a valuable predictive feature for gene essentiality in *E. coli *and *S. cerevisiae *[14,15]. Although protein-protein interaction networks may provide a global view of cellular signaling, we were rather interested in identifying drug targets in pathogens inferred from properties of mal-functional metabolism after having knocked out an enzymatic function.

In a previous study, we developed and applied an integrative machine learning method that combined these topology based methods to validate an experimental knock-out screen of *Escherichia coli *[16]. We now used the basic concepts of this strategy to enable predicting essential genes in an organism for which no experimental training data is available. For defining the essentiality of a gene, we now integrated sequence characteristics such as codon usages, length of the sequences and phyletic retention. Furthermore, we incorporated several centrality-descriptors for a node in a network. We used experimental datasets of comprehensive genome wide knock-out screens of *Escherichia coli *[17,18] and *Pseudomonas aeruginosa *[19,20] to train the machines with a large variety of attributes including topology characteristics as mentioned above, own developments on evaluating possible flux deviations [6], and genomic and transcriptomic information. To develop a classification system that is readily applicable for predicting essential genes of a new query organism, the system needs to make accurate predictions for an organism on which it was not trained. Therefore, we performed a cross-validation across the organisms of *E. coli *and *P. aeruginosa*, i.e. we trained with *E. coli *and validated with *P. aeruginosa *(and *vice versa*) to obtain the quality of the performance of this approach. We then applied the trained and validated classifiers to the pathogenic bacterium *Salmonella typhimurium*. We compared our results with the literature and experimental data of a large knock-out study for *S. typhimurium *[21]. Furthermore, we analyzed our predictions with gene set enrichment tests for metabolic pathways and identified proteins of the entire non-mevalonate pathway to be relevant for targeting with drugs. Its reactions showed typically topological characteristics of essential reactions. Using our prediction results and the experimental knock-out screen, we defined 35 enzymes as drug targets for *S. typhimurium*, 23 out of which have been described previously as drug targets in other micro-organisms. We suggest these and the remaining twelve as potential new drug targets for the organism we studied (*S. typhimurium*).

## Results and discussion

### Predicting essential genes with an organism-wise cross validation

**Figure 1:**
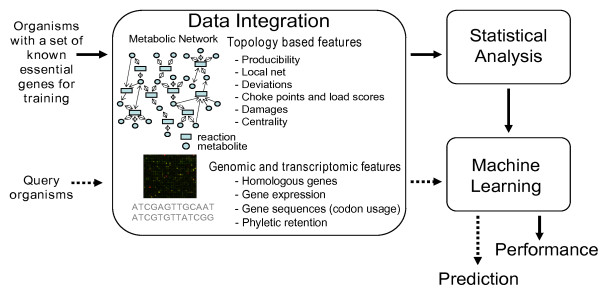
**The workflow**. Essentiality data for an organism was taken from an experimental genome wide knock-out screen. It was used to train the machine learning system using features that based on the topology of the metabolic network and genomic and transcriptomic data. The trained classifier was then applied to another organism (query organism) for which the essentiality for each gene in the metabolism was predicted.

**Figure 2:**
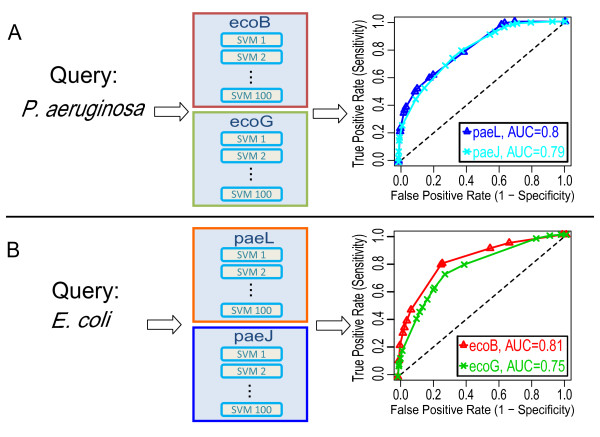
**ROC curves of the prediction performances**. (A) 100 Support Vector Machines were trained with the datasets ecoB and ecoG, respectively, and were then queried using the datasets from *P. aeruginosa *(union of the datasets paeL and paeJ). The number of machines predicting essentiality was summed up (voting score). Results from varying thresholds of the voting score were compared to the experimental results of paeL and paeJ yielding the ROC curves (area under the curve: 0.80 and 0.79, respectively). (B) Similar to (A) only that the machines were trained with the datasets of *P. aeruginosa *and queried with the datasets of *E. coli *resulting in ROC curves with AUC = 0.81 and 0.75 for the datasets ecoB and ecoG, respectively.

The general workflow is depicted in Figure 1. We reconstructed the metabolic networks for the investigated organisms by a bipartite graph consisting of alternating reactions and compounds. Two reactions were linked by a compound if the compound was a product of one reaction and a substrate of the other. The machine learning system was trained and validated with a large set of features. Local topology based features were used to qualitatively describe possible flux deviations. Choke and load points were defined and damage was used to describe the qualitative flux load and down stream effects of the knocked down reaction. Centrality features were calculated to additionally estimate the load of the reactions. We considered the existence of homologous genes for the corresponding knocked out genes which may be expected to take over the function. Co-regulated genes were considered to estimate the existence of possible analogous genes. Phyletic retention was calculated for observing phylogenetic conservation of the gene which was knocked out. Codon usages were calculated for each gene and used as features. All features are listed in Table 1 and are described in detail in the methods. We started predicting essential genes for *E. coli*. For this, we trained classifiers (machines) with the experimental data of two genome-wide knock-out screens of *Pseudomonas aeruginosa *(datasets paeJ and paeL from experimental studies of Jacobs and co-workers [19] and Liberati and co-workers [20], respectively). These datasets were taken as our gold standard defining true positives and true negatives of essential genes in the metabolism of the training organism (*P. aeruginosa*). We trained several (100) classifiers with all essential genes and an equal amount of randomly selected non-essential genes (stratification of the training data). The trained machines were then applied to predict essential genes for the query organism (*E. coli*). The output of all machines was summed up and used as a voting score that represented the propensity of a gene to be lethal for the cell. In turn, the same scheme was applied to predict essential genes for *P. aeruginosa *with classifiers which were now trained with two datasets from *E. coli *(ecoB from Baba and co-workers' study [17] and ecoG from Gerdes and co-workers' study [18], respectively). This organism-wise cross-validation was applied to estimate the performance of the classifiers. We compared the datasets for each genome. 79 of the essential genes were common in ecoB and ecoG, 92 were common in paeL and paeJ. One hundred machines were trained with different training-sets for each knock-out screen. Votes from both training sets for an organism were summed up and defined the stringency. A high number of votes for essentiality led to high specificity, while lower numbers led to higher sensitivity. The resulting receiver operator curves (ROC) of the classifiers are shown in Figure 2A for predicting *P. aeruginosa *and Figure 2B for predicting *E. coli*. For predicting essential genes for *P. aeruginosa *we yielded an area under the curve (AUC) of 0.80 and 0.79 when compared to the experimental datasets paeL and paeJ, respectively. In turn, for *E. coli *we yielded an AUC of 0.81 and 0.75 when compared to ecoB and ecoG, respectively. We wanted to obtain a reliable list of potential drug targets. For this, predictions for essential genes needed a low number of false positives. Hence, we set a high stringency and calculated the precision (true predictions out of all predictions for essentiality) with a high selection criterion (more than 195 out of 200 votes). We yielded a precision of 67% (accuracy: 87%, sensitivity: 7%, validating with paeL) and 100% (accuracy: 80%, sensitivity: 3%, validating with paeJ) when predicting essential genes for *P. aeruginosa*. In turn, we yielded a precision of 61% (accuracy: 87%, sensitivity: 27%, validating with ecoB) and 65% (accuracy: 80%, sensitivity: 18%, validating with ecoG) for *E. coli*. We yielded the best classifier results when using all features, in comparison to the classification performance when using individual sets of features (see Additional file 1: SupplementS1 for more details).

**Table 1:** List of all features.

Short form	Explanation
**Topology features**	
	**a) Deviation**
RUP	Reachable/Unreachable Products (RUP): equals one if all products could be produced when blocking the reaction, otherwise zero
PUP	Percentage of Unreachable Products (PUP): the percentage of products which cannot be produced when blocking the reaction
ND	Number of Deviations (ND)
APL	Average Path Length (APL): the average path length of the deviations
LSP	Length of the Shortest Path (LSP): the length of the shortest path of the deviations
	**b) Local topology**
NS	Number of Substrates (NS)
NP	Number of Products (NP)
NNR	Number of Neighboring Reactions (NNR)
NNNR	Number of Neighbors of Neighboring Reactions (NNNR)
CCV	Clustering Coefficient Value (CCV): clustering coefficient of a reaction
DIR	Directionality of a reaction (DIR)
	**c) Choke points and load scores**
CP	Choke Point (CP): a reaction is a choke point or not (Rahman *et al*, 2006)
LS	Load Score (LS): load score of a reaction (Rahman *et al*, 2006)
	**d) Damage**
NDR	Number of Damaged Reactions (NDR) (Lemke *et al*, 2004)
NDC	Number of Damaged Compounds (NDC) (Lemke *et al*, 2004)
NDRD	Number of Damaged Reactions having no Deviations (NDRD): the number of damaged reactions that have no other alternative paths to be reached after blocking a reaction
NDCD	Number of Damaged Compounds having no Deviations (NDCD): the number of damaged compounds that have no other alternative paths to be reached after blocking a reaction
NDCR	Number of Damaged Choke point Reactions (NDCR)
NDCC	Number of Damaged Choke point Compounds (NDCC)
NDCRD	Number of Damaged Choke point Reactions having no Deviations (NDCRD): the number of damaged choke point reactions that have no other alternative paths to be reached after blocking a reaction
NDCCD	Number of Damaged Choke point Compounds having no Deviations (NDCCD): the number of damaged choke point compounds that have no other alternative paths to be reached after blocking a reaction
	**e) Centrality**
BW	Betweenness centrality
CN	Closeness centrality
EC	Eccentricity centrality
EV	Eigenvector centrality
**Genomic and transcriptomic features**	
	**f) Homologs**
NAR	Number of Associated Reactions (NAR): the number of reactions that base on the knocked-out gene
Hn	Homology at different expectation values: the number of homologous genes with e-value cutoff 10^-30^,10^-20^,10^-10^,10^-7^,10^-5^,10^-3 ^(H30, H20, H10, H7, H5, H3)
	**g) Gene expression**
NGSE	Number of Genes having Similar Expression (NGSE): the number of genes that have similar expression (correlation coefficient >0.8)
MCC	Maximum of Correlation Coefficients (MCC): maximum value of the correlation coefficients for all neighboring genes
	**h) Phyletic retention**
PR	Phyletic Retention (PR): the number of orthologs in the other prokaryotes
	**i) Codon usage**
Nc	Number of codons
N3s	Base composition at silent sites (T3s, C3s, A3s, G3s)
glt	The frequency of amino acids glutamine (exemplarily)

### Examining the features

**Figure 3:**
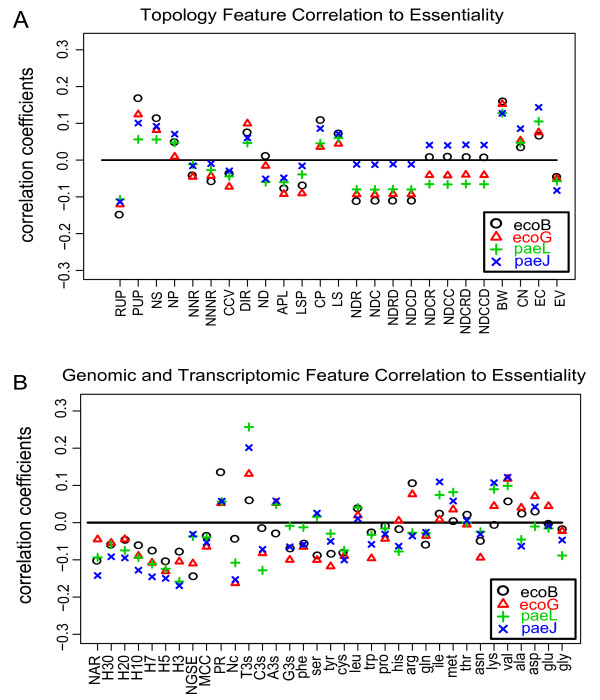
**Correlation of the features to essentiality**. The feature-values of each gene were correlated with the essentiality of the gene (1 = essential, 0 = non-essential). (A) shows the correlation coefficients for the topology features, (B) for the genomic and transcriptomic features. High values indicate that the feature was positively correlated to essentiality (see Additional file 2: SupplementS2 for all correlation coefficients). These values were obtained for all gold standards (ecoB, ecoG for *E. coli *and paeJ, paeL for *P. aeruginosa*).

We wanted to obtain an estimate of the correlations of our features to the essentiality of a gene. Therefore, we calculated Pearson's correlation coefficients of the essentiality class of each gene (1 = essential, 0 = non-essential) and the corresponding feature values. Figure 3 gives an overview for all features (see Additional file 2: SupplementS2 for correlation coefficients of all features). In the following, we describe the major results of our correlation study.

### Topology features

The efficiency of flux deviations was estimated by the features RUP and PUP which gave an estimate if all products of the knocked-out reaction could be produced without the reaction (RUP) and how large the percentage of non-producible products (PUP) was. RUP was a Boolean feature to observe if the mutant could produce all products of the knocked-out reaction. RUP = 1 was set if all downstream products could be produced by the mutant while RUP was set to zero if at least one downstream product could not be produced. The number of reachable products (RUP) was highly negative correlated and the percentage of unreachable products (PUP) highly positive correlated to the essentiality of a gene (P = 1.2E-10 and P = 2.4E-09, respectively) as shown in Figure 3A. If the (*in silico*) mutant could not produce one or more downstream products, RUP was zero whereas the percentage of unreachable products was increased in comparison to the situation in which all products could be produced. The higher the percentage of unreachable products of the mutant, the less products of the knocked out enzyme could be covered by alternative pathways. The number of substrates and products of the reactions of the knocked out gene (NS, NP) were positively correlated to gene essentiality (P = 4.3E-06 and P = 0.0172, respectively) showing that essential enzymes metabolize more different compounds. Interestingly, the number of neighboring reactions (NNR) and the number of neighbors of neighboring reactions (NNNR) showed a weak negative correlation to essentiality (P = 0.14 and P = 0.091, respectively). This is reasonable as a reaction with a high number of neighboring reactions may have more metabolites as products that can be produced by alternative enzymes. The clustering coefficients (CCV) showed the same tendency (negatively correlated, P = 0.018) also pointing to advantageous alternative pathways.

We estimated the feasibility of possible flux deviations by a set of features describing alternative pathways. The number of alternative pathways (ND), the average path length of the deviations (APL) and the length of the shortest alternative path (LSP) described the feasibility of alternative pathways. As expected, all of them were negatively correlated to essentiality (P = 0.15, P = 3.4E-04 and P = 0.0063, respectively), i.e. knocked out enzymes for which alternative pathways existed were less likely to cause a lethal phenotype if knocked out. Choke-points (CP) are uniquely consumed or produced compounds in the metabolism and showed a positive correlation with essentiality (P = 2.8E-04) as choke-points are often difficult to be replaced by the rest of the metabolism. Load-scores (LS) give an estimate of how often a reaction is involved in metabolic processes. They were also positively correlated to essentiality (P = 9.4E-04). Betweenness centrality (BW) and eccentricity (EC) were strongly positive correlated to essentiality (P = 1.3E-14 and 7.6E-08, respectively) showing that enzymes have a higher influence on vitality if placed in the center of the network. Closeness centrality (CN) also showed a positive correlation (P = 0.0020). Interestingly, the eigenvector centrality (EV) showed a negative correlation (P = 0.0013). Betweenness, closeness and eccentricity centrality are global centrality measures considering the whole network while the eigenvector centrality is a measure for local centrality and is computed from its neighbors. Note that typically a node with a high value of eigenvector centrality is a hub (node with high connectivity) with other hubs connected to it. Hence, flux deviations may be more likely for local hubs that have hubs in their vicinity making the node replaceable whereas global central nodes seemed to be generally substantial for maintaining the metabolic flow in the network. Therefore the eigenvector centrality may describe the network topology more in the sense as the clustering coefficient, specifically in respect to the likelihood of alternative pathways.

### Genomic and transcriptomic features

As expected, the number of homologous genes (H30, H20, H10, H7, H5, H3) showed a negative correlation to essentiality (P = 3.2E-04, 6.3E-04, 1.4E-06, 4.7E-09, 1.1E-10, 1.5E-09, respectively). Interestingly, an E-value cut-off of 10^-5 ^(H5) worked best showing that also non-perfectly matching sequences may take over functions of the knocked out gene. The number of genes having similar expression (NGSE) exhibited also a negative correlation to essentiality (P = 1.7E-04) which may be due to co-expression of genes with analogous function. For the feature phyletic retention (PR), the number of prokaryotes having orthologs of the knocked out gene showed a positive correlation to essentiality (P = 2.1E-16) supporting the findings of a previous study that conserved genes in evolution hint for their essentiality [14].

We analyzed the codon usage for each gene and related these to the essentiality of the gene. We found that genes with a high number of the nucleotide thymine at the third position of the codons were more likely to be essential for cell viability (feature T3s in Figure 3, see Additional file 3: SupplementS3 for histograms). The third codon position is the most redundant position in the genetic code. Matching of mRNA to tRNA codon nucleotides is less robust at the third position, and translational errors are therefore more likely to occur at that position. However, essential genes need to be stable and to be protected in the sequence. Thymine in the genetic code might cope for this as it was shown that thymine protected DNA and improved the efficiency of DNA replication [22]. Conserved genes are more likely to be essential [23] and a thymine at the 3^rd ^codon position facilitates stable genetic inheritance into off-springs and cellular replicates. Interestingly, we observed a larger difference of T3s in *E. coli *when compared to *P. aeruginosa*. It was found that a large average of G and C content at the third codon position is common for all genes in *P. aeruginosa *[24]. These results in a low T content at the third codon position which we observed and may explain the larger difference of T3s for essential and non-essential genes in *E. coli *compared to *P. aeruginosa *(see also Additional file 3: SupplementS3 for histograms).

Even though decision trees performed inferior compared to Support Vector Machines (data not shown), we used decision trees for an alternative approach to determine the most discriminating features for gene essentiality. Decision trees were trained with the datasets ecoB, ecoG, paeL and paeJ. It is known that the first decision of a decision tree is performed with the best discriminating feature [25]. Therefore, we counted the occurrence of our features at the top position of each run. Out of 400 runs, we got T3s (137×), BW (81×), Nc (38×), H3 (21×), H5 (15×), val (12×), H7 (11×), NGSE (11×), PUP (10×) as the first decision (features with less than 10 counts are not shown). This again showed that the feature for T3s substantially supported the classification.

### Identifying drug targets for *S. typhimurium*

We applied our trained machines from all four datasets (ecoB, ecoG, paeL, and paeJ) to predict essential genes for *S. typhimurium *and obtained votes from four hundred machines for each gene of *S. typhimurium *to be essential. To obtain a reasonable threshold for the number of votes predicting a gene to be essential, we compared the number of essentially predicted genes with the numbers of the training sets for *E. coli *and *P. aeruginosa*. For *E. coli *104 and 147 genes were essential corresponding to the datasets ecoB and ecoG, respectively, and for *P. aeruginosa*, 92 and 150 (corresponding to datasets paeL and paeJ, respectively). Therefore, we set a threshold of 350 votes (of 400 machines) to classify a gene as essential for *S. typhimurium *and obtained a comparable amount of 128 predicted essential genes. The complete list of genes being predicted as essential is given in the supplement (see Additional file 4: SupplementS4). We then compared our results to the experimental data from Knuth and co-workers who performed a large knock-out study for *S. typhimurium *[21]. They detected 6% of all open reading frames as being essential including 53 essential genes coding for enzymes in metabolism. For the remaining open reading frames of the genome they didn't make any prediction, including 711 genes for enzymes in metabolism. We compared the list of essential genes of Knuth and co-workers with our predictions and found 27 of our predicted genes in the list of Knuth and co-workers yielding a precision of 21%, an accuracy of 83% and sensitivity of 51%. It is to note that the experimental screen of Knuth and co-workers was not comprehensive; the authors stated in their article that for the genes not to be predicted as essential, they couldn't conclude that these genes are definitely non-essential. Therefore, our novel predictions may suit as potential new targets for further investigations. As a conservative and robust estimate of essential genes for *S. typhimurium*, we defined the corresponding enzymes of genes which were experimentally determined (by Knuth and co-workers) *and *were recognized by our classifiers. We then searched in the literature to find drug treatments of these enzymes for other micro-organisms. The results are listed in the following. Two asterisks (**) mark a gene for which enzyme we found clear evidence to be a drug target for a micro-organism. One asterisk (*) was set for a gene when we found reasonable evidence for its enzyme to serve as a drug target for a micro-organism. Hence, enzymes with one asterisk may serve for finding new drug targets and enzymes with two asterisks for transferring drug targets from other bacterial diseases to the disease we studied (*S. typhimurium*). Table 2 gives an overview of the results. We compared the open reading frames of the predicted genes with the human transcripts and did not detect significant homologs (using BLAST [26] and ENSEMBL cDNA transcripts [27]). E-values of the best hits are given in the supplement (see Additional file 5: SupplementS5). Here is the brief summary of the literature evidences we found:

**Table 2:** Predicted essential genes and potential drug targets.

ORF	Gene Symbol	EC	Enzyme	Evidence
**a) Intersection of our predictions with the experimental screen**				
STM0123	murE	6.3.2.13	UDP-N-acetylmuramoylalanyl-D-glutamate-2,6-diaminopimelate ligase	**
STM0128	murG	2.4.1.227	N-acetylglucosaminyl transferase	*
STM0129	murC	6.3.2.8	UDP-N-acetylmuramate-L-alanine ligase	**
STM0154	lpdA	1.8.1.4	Dihydrolipoamide dehydrogenase	
STM0218	pyrH	2.7.4.22	Uridylate kinase	*
STM0221	uppS	2.5.1.31	Undecaprenyl pyrophosphate synthase	**
STM0222	cdsA	2.7.7.41	CDP-diglyceride synthase	
STM0228	lpxA	2.3.1.129	UDP-N-acetylglucosamine acyltransferase	
STM0232	accA	6.4.1.2	Acetyl-CoA carboxylase	**
STM0489	hemH	4.99.1.1	Ferrochelatase	*
STM0535	lpxH		UDP-2,3-diacylglucosamine hydrolase	
STM0542	folD	1.5.1.5, 3.5.4.9	Bifunctional 5,10-methylene-tetrahydrofolate dehydrogenase	
STM0988	kdsB	2.7.7.38	CTP:CMP-KDO cytidylyltransferase	*
STM1194	fabD	2.3.1.39	Acyl carrier protein S-malonyltransferase	*
STM1195	fabG	1.1.1.100	3-ketoacyl-(acyl-carrier-protein) reductase	**
STM1200	tmk	2.7.4.9	Thymidylate kinase	
STM1700	fabI	1.3.1.10	Enoyl-(acyl carrier protein) reductase	
STM2483	dapE	3.5.1.18	Succinyl-diaminopimelate desuccinylase	
STM2652	pssA	2.7.8.8	Phosphatidylserine synthase	*
STM3090	metK	2.5.1.6		
STM3415	rpoA	2.7.7.6	DNA-directed RNA polymerase subunit alpha	
STM3724	kdtA		3-deoxy-D-manno-octulosonic-acid transferase	*
STM3730	dfp	4.1.1.36	Pantothenate kinase	**
STM3912	rep	3.6.1.-	ATP-dependent DNA helicase Rep	*
STM3978	yigC		3-octaprenyl-4-hydroxybenzoate decarboxylase	
STM4153	rpoB	2.7.7.6	DNA-directed RNA polymerase subunit beta	*
STM4154	rpoC	2.7.7.6	DNA-directed RNA polymerase subunit beta'	
**b) Predictions for the non-mevalonate pathway**				
STM0049	ispH, lytB	1.17.1.2	4-hydroxy-3-methylbut-2-enyl diphosphate reductase	*
STM0220	dxr	1.1.1.267	1-deoxy-D-xylulose 5-phosphate reductoisomerase	*
STM0422	dxs	2.2.1.7	1-deoxy-D-xylulose-5-phosphate synthase	**
STM0423	ispA	2.5.1.10	geranyltranstransferase	*
STM1779	ispE, ipk	2.7.1.149	4-diphosphocytidyl-2-C-methyl-D-erythritol kinase	**
STM2523	ispG, gcpE	1.17.7.1	4-hydroxy-3-methylbut-2-en-1-yl diphosphate synthase	*
STM2929	ispF	4.6.1.12	2-C-methyl-D-erythritol 2,4-cyclodiphosphate synthase	*
STM2930	ispD	2.7.7.60	2-C-methyl-D-erythritol 4-phosphate cytidylyltransferase	*

** clear evidence to be a drug target

* reasonable evidence to serve as a good drug target

** murE: UDP-N-acetylmuramoylalanyl-D-glutamate-2,6-diaminopimelate ligase is an essential enzyme and a well-known target against bacterial cell walls of *Staphylococcus aureus *[28].

* murG: N-acetylglucosaminyl transferase is a potential antibiotic targeting the biosynthesis of bacterial peptidoglycans. However, it is difficult to design inhibitors for this enzyme. Identifying inhibitors is under current research [29,30].

** murC: UDP-N-acetylmuramate-L-alanine ligase catalyzes an essential step in the pathway for synthesizing peptidoglycan precursors. Recently, new inhibitors of the MurC enzyme have been successfully tested for *Escherichia coli*, *Proteus mirabilis *and *Klebsiella pneumoniae *[31].

* pyrH: The gene for uridylate kinase is essential in *Mycobacterium tuberculosis *[32].

** uppS: Undecaprenyl pyrophosphate synthase (UPPS) is a novel antibacterial target of *Streptococcus pneumoniae *[33].

** accA: Acetyl-CoA carboxylase is a drug target for anti-obesity and antibiotic drugs [34,35].

* hemH: Ferrochelatase is essential for multiplication and intracellular survival of *Brucella abortus *[36].

* kdsB: Analogs of 3-deoxy-D-manno-octulosonate (KDO) were designed to inhibit CTP:CMP-KDO cytidylyltransferase (CMP-KDO synthetase) [37]. It is a potential target of *Haemophilus influenzae *[38] and *E.coli *[39].

* fabD: Acyl carrier protein S-malonyltransferase is a potential target of *Mycobacterium bovis BCG *[38].

** fabG: 3-ketoacyl-(acyl-carrier-protein) reductase is a well-known drug target of *E. coli, B. subtilis*, and *S. aureus *[40].

* pssA: Phosphatidylserine synthetase is required for motility and chemotaxis in *E. coli *[41]. Furthermore, mutants of *Escherichia coli *K12 which were defective in phosphatidylserine synthetase, were isolated as temperature-sensitive, conditional lethals [42].

* kdtA: 3-deoxy-D-manno-octulosonic-acid transferase is the enzyme of kdtA. In *E. coli*, it is essential for cell growth and accounts for conditional lethality associated with mutations in KDO biosynthesis [43].

** dfp: Pantothenate kinase is uptream of phosphopantothenoylcysteine decarboxylase in the biosynthesis of pantothenate and CoA. It is a well-known target for antimicrobial drugs against *E. coli *and *Mycobacterium tuberculosis *[44].

* rep: ATP-dependent DNA helicase Rep. Its deletion was found to be lethal in *B. subtilis *[45] and *Stapphylococcus aureus *[46].

* rpoB: DNA-directed RNA polymerase is a promising target for the discovery of new antimicrobial agents against *E. coli *[47].

### The non-mevalonate pathway and fatty acid biosynthesis are highly enriched with essential genes of *S. typhimurium*

**Figure 4:**
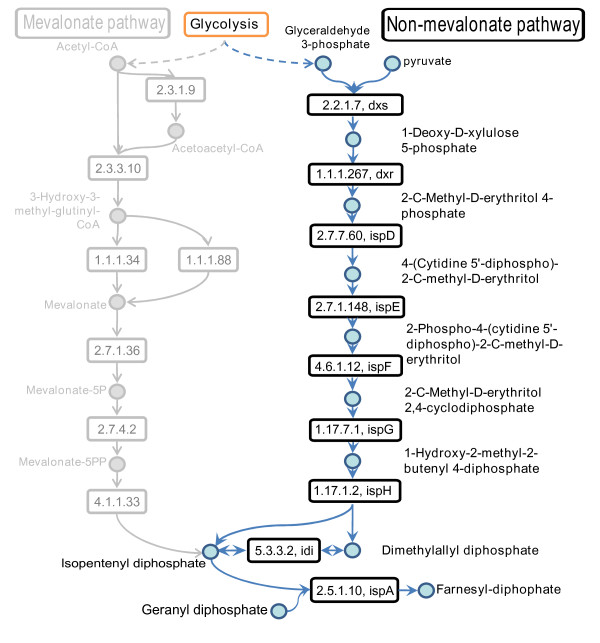
**The non-mevalonate pathway**. The non-mevalonate pathway produces isopentenyl diphosphate (IPP). It is an alternative pathway in bacteria and does not exist in the human host which uses the mevalonate pathway to produce IPP. The non-melavonate pathway was highly enriched with genes that were predicted to be essential. Reactions are given by their EC number and the gene symbol of the genes of the corresponding enzymes.

We performed gene set enrichment tests (Fisher's exact tests) with all pathways from KEGG [48] and found a significant enrichment of essential genes in the non-mevalonate pathway (P = 9.2E-06) and in the fatty acid biosynthesis pathway (P = 3.8E-04). Most of the genes in these pathways were essential (8 out of 9 genes in the non-mevalonate pathway and 8 out of 12 genes in the fatty acid biosynthesis pathway. The non-mevalonate pathway (Figure 4) produces isopentenyl diphosphate (IPP) and dimethylallyl pyrophosphate (DMAPP) that serve as a basis for the production of sterols, dolichols, and ubiquinone as well as components of macromolecules such as prenyl groups in proteins [49]. The pathway for non-mevalonate biosynthesis has been considered previously for attractive targets of novel antibiotics against bacteria [50,51] including *S. typhimurium *[52,53]. Figure 4 shows the non-mevalonate pathway and its essential enzymes for *S. typhimurium*. Note that the arrows in the figure do not represent information about the irreversibility of these reactions but rather show the direction of the overall flux. This rather linear pathway starts at 1-deoxy-D-xylulose-5-phosphate-synthase (EC 2.2.1.7) which corresponding gene dxs has been identified to be essential also by the experimental knock-out study of Knuth and co-workers [21]. The next six enzymes downstream were predicted to be essential by our method. The last enzyme we found in this pathway was geranyltranstransferase (EC 2.5.1.10). It catalyzes a reaction to produce farnesyl-diphosphate. Recently, Cornish and co-workers performed an elaborated mutagenesis study of the non-melavonate pathway in *S. typhimurium *and found five genes to be essential (ispD, ispE, ispF, ispG, ispH) [52]. We propose that all eight enzymes in this pathway are promising potential drug targets for *S. typhimurium*. We searched in the literature and indicated our findings by one and two asterisks as described above. For two genes we found clear evidences (two asterisks) and for six genes reasonable evidences (one asterisks) to code for drug targets (see Additional file 5: SupplementS5).

## Conclusions

We established a machine learning approach that predicts the essentiality of genes for an organism when no experimental knock-out data is available. The classifiers were trained with essentiality information for genes of one organism (e.g. *E. coli*) and were employed to predict essential genes of the other organism (e.g. *P. aeruginosa*). These predictions did not depend on essentiality information of the query organism for which the predictions were made, but solely on features that were calculated from the metabolic network and genomic and transcriptomic information of the query organism. Such data is abundantly available for many pathogenic bacteria. We applied this method to predict essential genes of *Salmonella typhimurium *as the query organism of interest and proposed 35 potential drug targets. 27 targets resulted from the intersection between our predictions and an experimental study [21] and 8 targets of the non-mevalonate pathway which we found by a statistical enrichment analysis. The non-mevalonate pathway is essential in algae, plants and several eubacteria including pathogenic bacteria. Enzymes of this pathway have been described to be potential targets for the development of novel antibiotics and herbicidal agents [50,52]. For *S. typhimurium*, we also inferred this by our machine learning approach. We discovered interesting correlations of our features to the essentiality of a gene. Various features describing the network topology served the machine to select reactions that showed no possible pathways for flux deviations, as e.g. in the linear non-mevalonate pathway. An intelligent combination of these features may be seen as an alternative approach to the established methods of flux balance analyses (FBA) and elementary flux modes (EFM) if detailed growth and nutrient information is lacking (which is needed for FBA, see [3] and if an in-depth refinement of the metabolic network is considered to be too labor intensive (in EFM the enzymes need to be separated into internal nodes and external nodes to reduce the computational complexity [54]).

Our method served well to estimate genes and their corresponding enzymes in the bacterial organisms of *E. coli*, *P. aeruginosa *and *S. typhimurium*. Inferring essentiality information for an organism from another organism may be facilitated by investigating a closely related organism as we did with *S. typhimurium *being rather closely related to *E. coli*. To apply this method to other micro-organisms, the metabolism may need to be well described, and the method may need adaptation for less studied organisms or which show special metabolic capabilities. For applying this method to eukaryotic genomes, the compartments in the cell at which a reaction occurs may need to be considered. It will be very challenging to apply our method for inferring multiple drug targets using experimental double knock-out screens as e.g. of the synthetic lethal project of eSGA [55]. For this, attributes that are related to single players (like e.g. sequence features) might be of less relevance while specifically network features might be much more related to synergistic knock-out effects. The challenge for the future remains to integrate such topological descriptive approaches with genetic information to systematically explore the network effects of enzyme treatments and combinations thereof.

## Methods

### Network reconstruction

The metabolic networks of *E. coli*, *P. aeruginosa*, and *S*. *typhimurium *were reconstructed using the database of KEGG [48]. Unspecific compounds such as water, ATP, etc. were discarded. Additionally, only the main compounds of the reactions as annotated in the KGML files of KEGG were used. Except for the centrality features and clustering coefficients, the topology features were calculated by a representation of the network as a bipartite graph consisting of metabolites and reactions as alternating nodes. For calculating the centrality features and the clustering coefficients, we represented the metabolic network as an undirected graph, also known as a reaction-pair network. It consisted of reactions as nodes and metabolites as edges connecting two reactions of the graph. Two reactions were connected by a metabolite if the metabolite was a product of one of the reactions and a substrate of the other reaction. Edges were discarded such that the network had no loops and no more than one edge between any two different nodes. Reactions were mapped to enzymes and enzymes mapped to their corresponding genes using the association tables from KEGG. Genes that corresponded to death-end reactions in the network were not included into the datasets for training and validation. If a gene corresponded to more than one reaction, the mean value of the reaction features was taken. For the Boolean features (RUP, DIR, CP, see below) we used the Boolean OR-operation, i.e. a gene feature was set to one if at least one reaction feature equaled to one.

### The gold standards

To train and validate our predictions, we used published datasets from genome wide experimental knock-out screens. All were performed in LB rich medium. Two datasets were of *E. coli *and two of *P. aeruginosa*. Additionally we used data from a knock-out study of *S. typhimurium *which was also performed in LB rich medium. All datasets were taken from the NMPDR database [56]. For *E. coli*, we used the KEIO collection of Baba and co-workers [17] which we denoted as 'ecoB'. It consisted of 104 essential and 641 non-essential genes for the metabolic network. The other dataset of *E. coli *was from Gerdes and co-workers [18] which we denoted as 'ecoG'. It consisted of 147 essential genes and 533 non-essential genes for our network. For *P. aeruginosa *we used the data of Liberati et al. [20] denoted as 'paeL'. It consisted of 92 essential genes and 615 non-essential genes for the network. The other dataset for *P. aeruginosa *was taken from the study by of Jacobs et al. [19]. We denoted it as 'paeJ'. It consisted of 150 essential genes and 579 non-essential genes. The experimental dataset for *S. typhimurium *was from Knuth and co-workers [21] and based on insertion-duplication mutagenesis (IDM). Small, randomly generated genomic fragments were cloned into a conditionally replicating vector, and the resulting library of single *S. typhimurium *clones was grown under permissive conditions. Upon switching to non-permissive temperature, discrimination between lethal and non-lethal insertions following homologous recombination allowed the trapping of genes with essential functions. With this method, genes were detected that were indispensable for growth. However, non-essential genes could not be determined. For the metabolism, 53 genes were found to be essential and for the remaining 711 the essentiality could not be determined by this method [21].

### Defining the features

Features were obtained from network topology properties and genomic and transcriptomic information. Table 1 shows an overview of all features and their abbreviations.

### Topology based features

#### a) Deviation features

For the following features, we used the metabolic network in the representation of a bipartite graph consisting of two different alternating nodes, i.e. metabolites and reactions. As reported recently [6,16], we implemented a breadth first algorithm to investigate the network when a single reaction was blocked. We defined a reaction as essential for survival when basically the mutated network could not yield the products of the reaction from upstream substrates of the reaction. Hence, features were defined to describe if the knocked out reaction was substantial for producing its downstream metabolites or if these products could still be produced by other pathways. The investigation for each tested knocked out reaction was performed by the following algorithm:

i. All metabolites acting as input nodes (substrates) and output nodes (products) of the knocked out reaction were selected. The set of substrates S defined the input nodes and the set of products P defined the output nodes. To get a broader list of available substrates we integrated other substrates into S. We included the substrates of the upstream reactions and the products of the downstream reactions into the sets S and P, respectively. Substrates of reactions that had at least one of the substrates S as a substrate were included into S. Further, substrates of reactions that had a metabolite out of P as a substrate were also included into S.

ii. Reactions were selected which used only available compounds as substrates.

iii. These selected reactions and their products were incorporated into the list of discovered reactions and products. The products were set as newly available metabolites in the network.

iv. Steps ii and iii were repeated until no further reactions could be identified.

v. The output nodes that could be produced were counted (reachable products P).

After finishing the process, we used the number of defined output nodes that could be produced within the mutated network for two features, i.e. a quality feature defining if all products could be produced (RUP, reachable/unreachable products), and the percentage of products that could not be produced (PUP, percentage of unreachable products). We again run a breadth first search on the network to estimate possible deviations. Starting from S, the breadth first search explored the network for finding the direct products of the knocked out reaction. When the algorithm visited these products, it stored the corresponding pathway and continued its search to find further alternative paths until the network was entirely explored or a maximal path length of 10 reactions was reached. The organism may have many pathways to produce the products making the system more robust. Thus, we counted the number of possible alternative paths yielding feature ND (ND, number of deviations). We took the average path length (APL, average path length) and the shortest path length (LSP, length of shortest path) of the deviations as features for the classifier. The deviation features were used to find alternative pathways to produce products of the knocked out reaction by its substrates S. In the metabolic network, these substrates can also be consumed by other reactions yielding their products etc. Therefore, we kept track of alternative paths in the network for the potential of the organism to survive when a reaction was blocked.

#### b) Local topology

The number of substrates and products of the knocked out reaction were counted (NS: number of substrates; NP: number of products). We defined features for the number of Neighboring reactions (NNR) and the number of Neighbors of Neighboring reactions (NNNR). We calculated the clustering coefficient (CCV) as described in [57,58] of the knocked out reaction to estimate the local density of the network. The reaction direction (DIR, directionality of reactions) was taken from KEGG and set as reversible if no other information was available.

#### c) Choke-points and load-scores

A reaction that uniquely consumes or produces a certain metabolite in the metabolic network is considered a choke point. Such a reaction shows high potential for essentiality [4,5]. We used this as a feature (CP, choke points). According to the concept [4], load scores were defined as hot spots in the network based on the ratio of the number of k-shortest paths passing through a reaction, and the number of nearest neighbor links attached to it. This ratio was compared to the average load value in the network.

#### d) Damage

The damage was defined to determine potentially effected metabolites and reactions downstream of the knocked out reaction. We used the definition of damaged compounds and reactions from Lemke and co-workers [7] yielding the features NDR (NDR, number of damaged reactions) and NDC (number of damaged compounds). As half of our reactions were annotated as being reversible, some compounds and reactions might have been inferred as damage but are actually just back-traced alternative pathways. Therefore, we calculated the number of damaged compounds and reactions for a network in which all alternative pathways were discarded yielding NDRD (number of damaged reactions without deviations) and NDCD (number of damaged compounds without deviations). In addition, we calculated the number of damaged choke points (NDCR, number of damaged choke point reactions; NDCC, number of damaged choke point compounds; NDCRD, number of damaged choke point reactions without deviations; NDCCD, number of damaged choke point compounds without deviations).

#### e) Centrality

For these features, we used the network-representation of a reaction-pair network. We computed the centrality features by using the R package 'igraph' [59] consisting of betweenness centrality (BC), closeness centrality (CN), eccentricity (EC) [10], and eigenvector centrality (EV) [60]. Let G(*V, E*) be a simple undirected graph with *n *vertices (reactions). Betweenness measures the frequency of a reaction (node) to be in the shortest path of all pairs of reactions [10]. The betweenness centrality *C*_*b*_(*v*) for a reaction *v *is given by(1)

in which *d*_*ij *_is the number of shortest paths from reaction *i *to reaction *j*, and *d*_*ij*_*(v) *is the number of shortest paths from *i *to *j *that pass through reaction *v*. The sum is composed of all pairs (*i, j*) of reactions of the network. Closeness centrality approximates how many edges are required to access every other reaction from a given reaction [10]. It is defined by the inverse of the average length of the shortest paths to all the other reactions. The closeness centrality *C*_*c*_(*v*) for a reaction *v *is given by(2)

in which *n *is the number of reactions in the network. Eccentricity is the longest distance from the given reaction to any other reaction [61]. The eccentricity *C*_*e*_(*v*) for a vertex *v *is given by(3)

Eigenvector centrality is based on the assumption that the utility of a reaction is determined by the utility of the neighboring reactions [62]. It scores a reaction higher if it is connected to high-scoring reactions. It is defined as the principal eigenvector of the adjacency matrix of the network. Let *x*_*i *_denote the score of a reaction *i*. Let *A*_*ij *_be the adjacency matrix of the network. Thus *A*_*ij *_= 1 if there is an edge between reactions *i *and *j*, and *A*_*ij *_= 0 otherwise. For reaction *i*, the centrality score is proportional to the average of the centralities of *i*'s network neighbors:(4)

in which *Neighbor(i) *is the set of neighboring reactions of reaction *i*, *n *is the total number of reactions and λ is a constant. This leads directly to the well-known eigenvector equation, Ax = λx. Normally, there are different eigenvalues λ for which an eigenvector solution exists. According to the Perron-Frobenius theorem only the eigenvector of the largest eigenvalue is the eigenvector centrality [60].

### Genomic and transcriptomic features

#### f) Homologs

We calculated the number of homologous genes that might have taken over the function of the knocked out gene. Homologous genes were searched using BLAST [26] against all open reading frames of the respective organism (*E. coli*, *P. aeruginosa*, *S. typhimurium*). We used different E-value cutoffs, i.e. 10^-3^, 10^-5^, 10^-7^, 10^-10^, 10^-20^, and 10^-30 ^to obtain the features H3, H5, H7, H10, H20 and H30, respectively. Sequences of all open reading frames were taken from the NCBI database (http://www.ncbi.nlm.nih.gov/, *E. coli*: [GenBank:NC_000913], *P. aeruginosa*: [GenBank:NC_002516], and *S. typhimurium*: [GenBank:NC_003197]).

#### g) Gene expression

We collected gene expression data for all three investigated organisms from public resources. The datasets were selected in respect to have a rather unspecific regulation, i.e. from treatments affecting not a small band but a broad range of metabolic pathways. For *E. coli*, we used gene expression data from a study in which the regulation during oxygen deprivation was investigated [63], for *P. aeruginosa *from a study observing the response to agmatine and putrescine treatment [64] and from a study of quorum-sensing response to environmental conditions [65]. For *S. typhimurium *we used data of cells treated with nutrient limitation at different time points [66] and data from a study that captured the regulatory response in the environment of the host [67]. The data was normalized by variance stabilization normalization [68]. Genes with similar functionality in the same pathway often show co-regulation [69]. Therefore, the maximum correlation coefficient (MCC) of all neighboring reactions of the knocked out reaction was used as a feature. Additionally, we calculated the number of reactions with similar gene expression (NGSE, correlation coefficient > 0.8) and used it as features for an estimate of co-regulated analogous genes.

#### h) Phyletic retention

We selected 177 prokaryotic organisms (except *E. coli*, *P. aeruginos*a, and *S. typhimurium*) as described in Gustafson *et al*. [14] out of which we counted the number of organisms having an open reading frame that was homologous the sequence of the knocked out gene. This was performed with *E. coli*, *P. aeruginosa*, and *S. typhimurium *using bi-directional best BLAST hits (E-value cutoff of 0.1).

#### i) Codon usage

Codons were counted for each investigated gene from its coding region. We counted base compositions at silent sites (third position of the codons) yielding the features T3s, C3s, A3s, G3s for thymine, cytosine, adenine and guanine, respectively. Additionally, the number of codons coding for all encoded amino acids (phe, ser, tyr, cys, leu, trp, pro, his, arg, gln, ile, met, thr, asn, lys, val, ala, asp, glu, gly) were counted. All codon counts were normalized by division of the total number of codons (Nc). Nc was also used as a feature.

### The machine learning system

We used Support Vector Machines from the R package 'e1071' to classify essential and non-essential genes of metabolism http://www.r-project.org. A radial basis function was used as the kernel. Parameter optimization was performed on the training data for the regularization term and the kernel width. The regularization term defined the costs for false classifications and was optimized using the values 2^*n *^with *n *= -4, -2, 0, 2, 4. The same range was taken for the kernel width. This optimization was realized by training with a grid search over all combinations of these parameters. The sizes of the two classes differed considerably in our data sets (essential genes: 8 - 15%, non-essential genes: 85 - 92%). For a broad spectrum of different sensitivities and specificities, we applied a voting scheme. We trained 100 Support Vector Machines (SVMs) with all essential genes and an equal amount of randomly selected non-essential genes. With this, we stratified the training data. For the classification of a query gene, the output of all machines was summed up and used as a voting score for the gene to be essential for the cell.

### Defining the most discriminating features with decision trees

The first decision in decision trees applies the best discriminating feature [25]. Therefore, we used decision trees as an alternative approach for defining the most discriminating features. We applied the method of decision trees using the R package 'rpart' http://www.r-project.org to classify essential and non-essential genes of the metabolism. Gini impurity [70] was used for splitting the data. The minimum number of observations that had to exist in a node was 20 and the maximum depth was 30. We stratified the training data in the same manner as for training the Support Vector Machines. One hundred decision trees were generated for each gold standard (ecoB, ecoG, paeL and paeJ). To get the most discriminating features, for each run the first decision of each tree was selected.

### Performance measures and statistics

For assessing the performance of the classifier, the predictions were compared to the gold standard from the experimental screens. A prediction was either true positive (tp, prediction: essential, gold standard: essential), false positive (fp, prediction: essential, gold standard: non-essential), true negative (tn, prediction: non-essential, gold standard: non-essential) or false negative (fn, prediction: non-essential, gold standard: essential). We calculated the standard measures accuracy = (tp+tn)/(tp+tn+fp+fn), sensitivity = tp/(tp+fn), and specificity = tn/(tn+fp). A receiver operator characteristics (ROC-curve) was used to measure the performance for a classifier system with various thresholds. In the ROC-curve the sensitivity is plotted against 1 - specificity and the area under the curve (AUC) yields a performance estimate across the entire range of thresholds. P-values for Pearson's correlation coefficients for the features were calculated as described in [71].

## Authors' contributions

KP and RK put up the general concept and design of the study. KP carried out the data analysis. KP and RK drafted the manuscript. All authors read and approved the final manuscript.

## Supplementary Material

Additional file 1 ROC curves for the essential gene predictions with subsets of features. Click here for file

Additional file 2 Correlation coefficients of the features to gene essentiality. Click here for file

Additional file 3 Histograms for the frequency of T3s in essential genes and non-essential genes. Click here for file

Additional file 4 All examined metabolic genes of *S. typhimurium *with our computational predictions and the experimental results of Knuth et al. (2004). Click here for file

Additional file 5 Predicted essential genes and potential drug targets for *S. typhimurium *and their literature evidences. Click here for file
