# Supplementary material for: Identifying essential genes in bacterial metabolic networks with machine learning methods
Source: BMC Syst Biol. 2010 May 3;4:56. doi: 10.1186/1752-0509-4-56 (PMC2874528; doi:10.1186/1752-0509-4-56)
Supplement: Additional file 1 — ROC curves for the essential gene predictions with subsets of features. [file 1752-0509-4-56-S1.PDF]

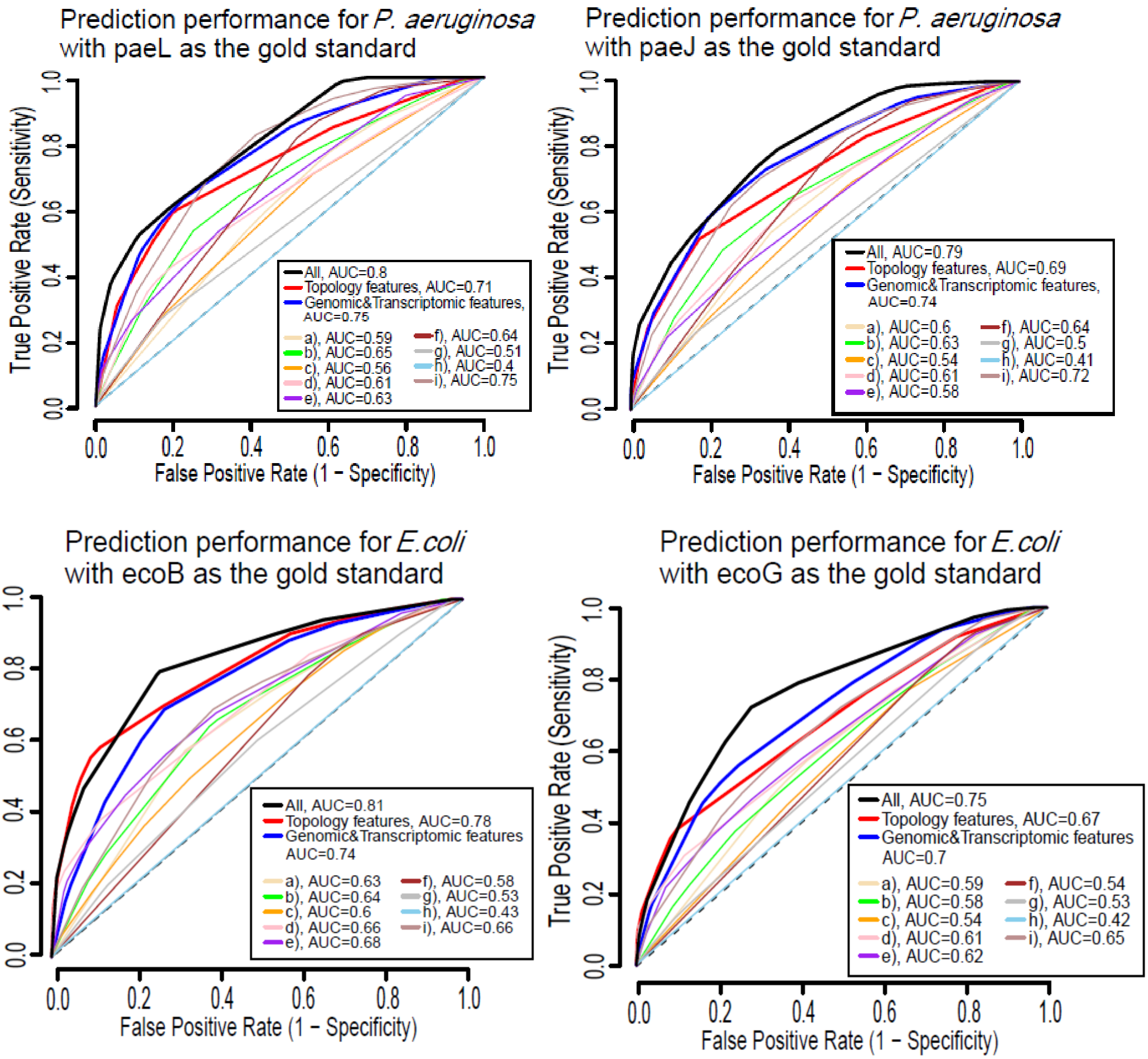

Figure S1. ROC curves for the essential gene predictions with subsets of features.

To evaluate the performance of different subsets of our features, we trained the machines with subsets of features according to their basic groupings (feature groupings, see Table 1) to predict essential genes. The figure shows their performances for *P. aeruginosa* by using *E. coli* for training (upper row, the left (right) diagram shows the performances with paeL (paeJ) as the gold standard) and vice versa (lower row, the left (right) diagram shows the performances with ecoB (ecoG) as the gold standard). To estimate the overall performances, we calculated the area under the curves (AUC, see figure inserts). The machines that used all features performed best (black curves) followed by the set of all topology features.
