# Supplementary material for: Identifying essential genes in bacterial metabolic networks with machine learning methods
Source: BMC Syst Biol. 2010 May 3;4:56. doi: 10.1186/1752-0509-4-56 (PMC2874528; doi:10.1186/1752-0509-4-56)
Supplement: Additional file 3 — Histograms for the frequency of T3s in essential genes and non-essential genes. [file 1752-0509-4-56-S3.PDF]

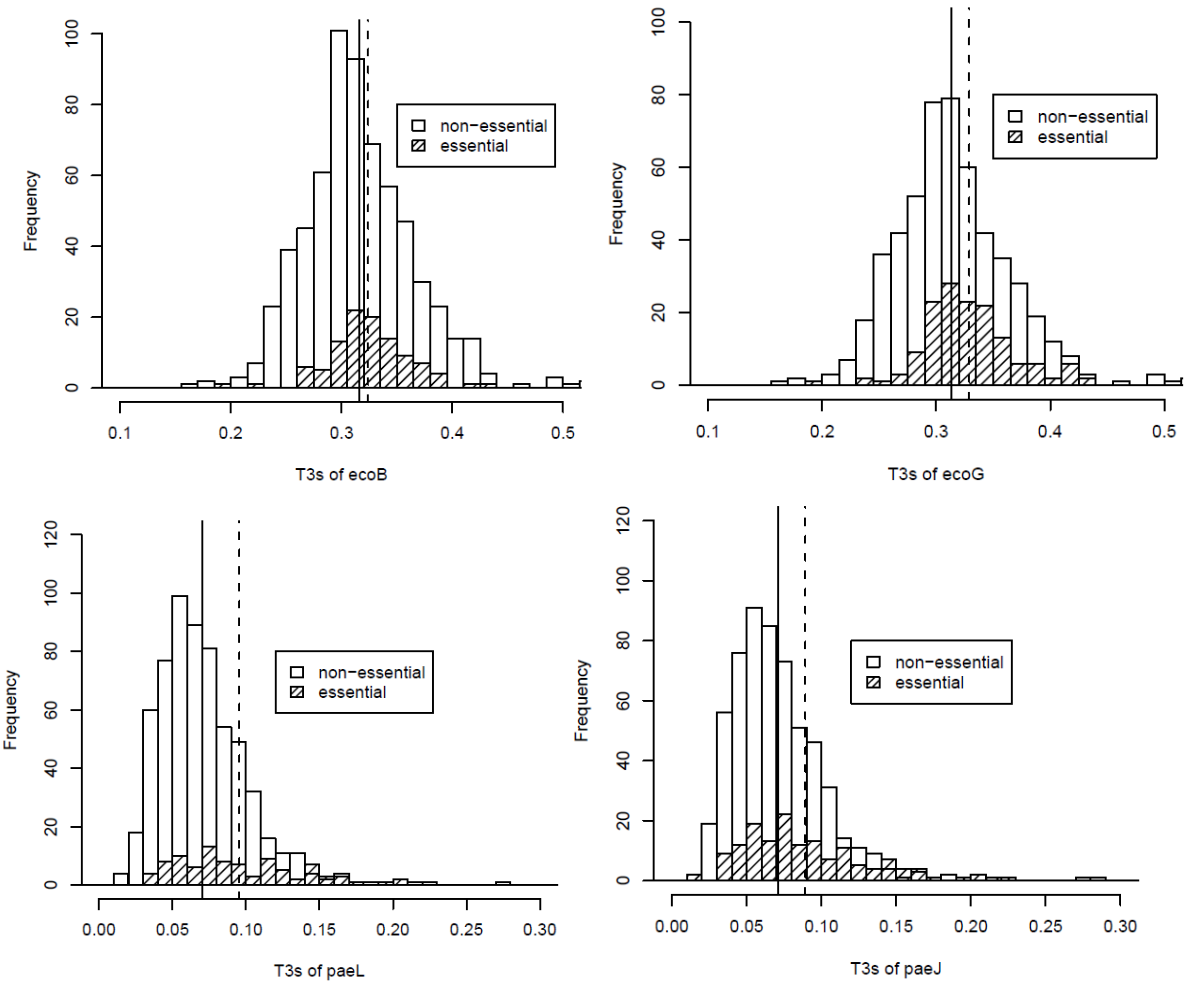

Figure S3. Histograms for the frequency of T3s in essential genes and non-essential genes of *E. coli* (upper row) and *P. aeruginosa* (lower row)

To examine the relationship between the number of thymines at the 3rd codon position (T3s) and gene essentiality, the figures show T3s distributions of essential and non-essential genes in *E. coli* (upper row, left and right for the datasets ecoB and ecoG, respectively) and in *P. aeruginosa* (lower row, left and right for the datsets paeL and paeJ, respectively). Dashed lines indicate the average of T3s in essential genes, solid lines indicate the average of T3s in non-essential genes. *E. coli* and *P. aeruginosa* are gamma-proteobacteria which are not closely related. It has been observed that *P. aeruginosa*'s genome is GC-rich while *E. coli's* genome shows an ordinary GC content. The large average of G and C at the third codon position is common for all genes in *P. aeruginosa* [1]. This results in a low T content of the third codon position which we also can observe here and may explain the larger difference of T3s for essential and non-essential genes in *E. coli* compared to *P.aeruginosa*.

Reference

1. Grocock RJ, Sharp PM: **Synonymous codon usage in Pseudomonas aeruginosa PA01**. *Gene* 2002, **289**:131-139.
