# Supplementary material for: Identifying essential genes in bacterial metabolic networks with machine learning methods
Source: BMC Syst Biol. 2010 May 3;4:56. doi: 10.1186/1752-0509-4-56 (PMC2874528; doi:10.1186/1752-0509-4-56)
Supplement: Additional file 5 — Predicted essential genes and potential drug targets for S. typhimurium and their literature evidences. [file 1752-0509-4-56-S5.PDF]

Supplement Table S5. Predicted essential genes and potential drug targets for *S. tyhpimurium* (a detailed description is given below the table)

ORF id	Gene Symbol	EC number	Enzyme	Evidence	Human homologs	E value
**a) Intersection of our predictions with the experimental screen**						
STM0123	murE	6.3.2.13	UDP-N-acetylmuramoylalanyl-D-glutamate-2,6-diaminopimelate ligase	**	ENST00000364688	9.6
STM0128	murG	2.4.1.227	N-acetylglucosaminyl transferase	*	ENST00000408249	0.73
STM0129	murC	6.3.2.8	UDP-N-acetylmuramate-L-alanine ligase	**	ENST00000408663	0.61
STM0154	lpdA	1.8.1.4	Dihydrolipoamide dehydrogenase		ENST00000411150	2.1
STM0218	pyrH	2.7.4.22	Uridylate kinase	*	ENST00000410942	4.7
STM0221	uppS	2.5.1.31	Undecaprenyl pyrophosphate synthase	**	ENST00000386578	0.18
STM0222	cdsA	2.7.7.41	CDP-diglyceride synthase		ENST00000362327	5.5
STM0228	lpxA	2.3.1.129	UDP-N-acetylglucosamine acyltransferase		ENST00000386484	1.3
STM0232	accA	6.4.1.2	Acetyl-CoA carboxylase	**	ENST00000410499	6.1
STM0489	hemH	4.99.1.1	Ferrochelatase	*	no hit	
STM0535	lpxH		UDP-2,3-diacylglucosamine hydrolase		ENST00000386574	4.6
STM0542	folD	1.5.1.5, 3.5.4.9	Bifunctional 5,10-methylene-tetrahydrofolate dehydrogenase		no hit	
STM0988	kdsB	2.7.7.38	CTP:CMP-KDO cytidylyltransferase	*	ENST00000386088	1.2
STM1194	fabD	2.3.1.39	Acyl carrier protein S-malonyltransferase	*	ENST00000385201	5.9
STM1195	fabG	1.1.1.100	3-ketoacyl-(acyl-carrier-protein) reductase	**	ENST00000388337	0.3
STM1200	tmk	2.7.4.9	Thymidylate kinase		ENST00000387015	4.1
STM1700	fabI	1.3.1.10	Enoyl-(acyl carrier protein) reductase		ENST00000387331	1.3
STM2483	dapE	3.5.1.18	Succinyl-diaminopimelate desuccinylase		ENST00000408717	7.2
STM2652	pssA	2.7.8.8	Phosphatidylserine synthase	*	ENST00000365512	8.7
STM3090	metK	2.5.1.6			ENST00000388372	7.4
STM3415	rpoA	2.7.7.6	DNA-directed RNA polymerase subunit alpha		ENST00000385068	1.1
STM3724	kdtA		3-deoxy-D-manno-octulosonic-acid transferase	*	ENST00000363352	2.1
STM3730	dfp	4.1.1.36	Pantothenate kinase	**	ENST00000410954	0.5
STM3912	rep	3.6.1.-	ATP-dependent DNA helicase Rep	*	ENST00000222567	0.2
STM3978	yigC				ENST00000364285	0.61
STM4153	rpoB	2.7.7.6	DNA-directed RNA polymerase subunit beta	*	ENST00000362682	6.5
STM4154	rpoC	2.7.7.6	DNA-directed RNA polymerase subunit beta'		ENST00000388141	1.7

b) Predictions for the non-mevalonate pathway

STM0049	ispH, lytB	1.17.1.2	4-hydroxy-3-methylbut-2-enyl diphosphate reductase	*	ENST00000408450	0.41
STM0220	dxr	1.1.1.267	1-deoxy-D-xylulose 5-phosphate reductoisomerase	*	ENST00000384092	2
STM0422	dxs	2.2.1.7	1-deoxy-D-xylulose-5-phosphate synthase	**	ENST00000387061	3
STM0423	ispA	2.5.1.10	geranyltranstransferase	*	ENST00000410444	1.3
STM1779	ispE, ipk	2.7.1.149	4-diphosphocytidyl-2-C-methyl-D-erythritol kinase	**	ENST00000386764	5.5
STM2523	ispG, gcpE	1.17.7.1	4-hydroxy-3-methylbut-2-en-1-yl diphosphate synthase	*	ENST00000410400	7.2
STM2929	ispF	4.6.1.12	2-C-methyl-D-erythritol 2,4-cyclodiphosphate synthase	*	ENST00000384847	12
STM2930	ispD	2.7.7.60	2-C-methyl-D-erythritol 4-phosphate cytidylyltransferase	*	ENST00000364025	0.07

** clear evidence to be a drug target

* reasonable evidence to serve as a good drug target

Detailed description of the predicted drug targets

We defined 27 enzymes as promising drug targets by intersecting our predictions with the results from the experimental screen (and inferring the enzymes from the corresponding coding genes). An overview is given in Table S5-a. Additionally, we predicted 8 out of 9 genes from the non-melavonate pathway. These genes are listed in Table S5-b. We compared the open reading frames of the predicted genes with the human transcripts and did not detect significant homologs (using BLAST [1] and ENSEMBL cDNA transcripts [2]). E-values of the best hits and the best hits are given in the last two columns of Table S5.

We searched through the literature to compare our results with treatments against other micro-organisms. Two asterisks (**) mark a gene for which enzyme we found clear evidence to be a drug target for a micro-organism. One asterisk (*) was set for a gene when we found reasonable evidence for its enzyme to serve as a good drug target for a micro-organism. Hence, enzymes with one asterisk may serve for finding new drug targets and enzymes with two asterisks for transferring drug targets from other diseases to the disease we studied (*S. typhimurium*). Astericks, gene ids, gene symbols and enzyme commission numbers are given.

a) Literature evidences for drug targets defined by intersecting our predictions with the experimental knock out screen:

** STM0123 murE EC 6.3.2.13: UDP-N-acetylmuramoylalanyl-D-glutamate-2,6-diaminopimelate ligase is an essential enzyme and a well-known target against bacterial cell walls of *Staphylococcus aureus* [3].

* STM0128 murG EC 2.4.1.227: N-acetylglucosaminyl transferase is a potential antibiotic targeting the biosynthesis of bacterial peptidoglycan. However, it is difficult to design inhibitors for this enzyme. Identifying inhibitors is under current research [4, 5].

** STM0129 murC EC 6.3.2.8: UDP-N-acetylmuramate-L-alanine ligase catalyzes an essential step in the pathway for synthesizing peptidoglycan precursors. Recently, new inhibitors of the MurC enzyme have been successfully tested for *Escherichia coli*, *Proteus mirabilis* and *Klebsiella pneumoniae* [6].

* STM0218 pyrH EC 2.7.4.22: Uridylate kinase is an essential gene for *Mycobacterium tuberculosis* [7].

** STM0221 uppS EC 2.5.1.31: Undecaprenyl pyrophosphate synthase (UPPS) is a novel antibacterial target in *Streptococcus pneumoniae* [8].

** STM0232 accA EC 6.4.1.2: Acetyl-CoA carboxylase is a drug target for anti-obesity and antibiotic drugs [9, 10].

* STM0489 hemH EC 4.99.1.1: Ferrochelatase is essential for multiplication and intracellular survival of *Brucella abortus* [11].

* STM0988 kdsB EC 2.7.7.38: Analogs of 3-deoxy-D-manno-octulosonate (KDO) were designed to inhibit CTP:CMP-KDO cytidylyltransferase (CMP-KDO synthetase)[12]. It is a potential target for *Haemophilus influenzae* [13] and *E.coli* [14].

* STM1194 fabD 2.3.1.39: Acyl carrier protein S-malonyltransferase is a potential target against *Mycobacterium bovis BCG* [15].

** STM1195 fabG EC 1.1.1.100: 3-ketoacyl-(acyl-carrier-protein) reductase is a well-known drug target against micro-organisms including *E. coli, B. subtilis*, and *S. aureus* [16].

* STM2652 pssA EC 2.7.8.8: Phosphatidylserine synthetase is required for motility and chemotaxis in *E. coli* [17]. Furthermore, mutants of *Escherichia coli* K12 which were defective in phosphatidylserine synthetase, were isolated as temperature-sensitive, conditional lethals [18].

* STM3724 kdtA: 3-deoxy-D-manno-octulosonic-acid transferase is encoded by kdtA. In *E. coli,* it is essential for cell growth and accounts for conditional lethality associated with mutations in KDO biosynthesis [19].

** STM3730 dfp EC 4.1.1.36: Pantothenate kinase is uptream of phosphopantothenoylcysteine decarboxylase in the pathway for biosynthesis of pantothenate and CoA. It is a well-known target for antimicrobial drugs against *E. coli* and *Mycobacterium tuberculosis* [20].

* STM3912 rep EC 3.6.1.-: ATP-dependent DNA helicase Rep. Its deletion was found to be lethal in *B. subtilis* [21] and *Stapphylococcus aureus* [22].

* STM4153 rpoB EC 2.7.7.6: DNA-directed RNA polymerase is a promising target for the discovery of new antimicrobial agents against *E. coli* [23].

b) Literature evidences for the non-mevalonate pathway:

* STM0049 ispH (lytB) EC 1.17.1.2: Lethal mutation of ispH and other genes in the MEP pathway were found in *S. typhimurium* [24].

* STM0220 dxr EC 1.1.1.267: 1-Deoxy-D-xylulose 5-phosphate reductoisomerase (DXR) is a key enzyme of the MEP pathway for *Bacillus anthracis*, *Helicobacter pylori*, *Yersinia pestis*, *Mycobacterium tuberculosis* and the malaria parasite *Plasmodium falciparum.* It was reported to be a rational target for drug design [25].

** STM0422 dxs EC 2.5.1.31: 1-deoxy-D-xylulose-5-phosphate synthase (DXS) is a known target against *Mycobacterium tuberculosis* [26].

* STM0423 ispA EC 2.5.1.10: It was shown that farnesyl pyrophosphate synthase (geranyltranstransferase) is a potential chemotherapeutic target against parasitic protozoans [27].

** STM1779 ispE (ipk) EC 2.7.1.148: 4-Diphosphocytidyl-2C-methyl-D-erythritol kinase (IspE) is a target for anti-infective drugs which was tested for *E. coli* and *Aquifex aeolicus* [28, 29].

* STM2523 ispG (gcpE) EC 1.17.7.1: The enzyme 4-hydroxy-3-methylbut-2-en-1-yl diphosphate synthase is the last step in the pathway. It produces 2-C-Methyl-D-erythritol 2,4,cyclodiphosphate which is then converted to isopentenyl-PP. It was reported that this enzyme is a reasonable target for anti-infection [30].

* STM2929 ispF EC 4.6.1.12 and STM2930 ispD EC 2.7.7.60: All enzymes of the non-mevalonate pathway including 2C-methyl-D-erythriol 2,4-cyclodiphosphate synthase and 4-diphosphocytidyl-2C-methyl-D-erythriol synthase from *E. coli* were successfully screened with large compound libraries [28].

Lethal mutations were found in independent mutations of ispD, ispE, ispF, ispG and ispH in *S. typhimurium* [24].

References

1. Altschul SF, Madden TL, Schaffer AA, Zhang J, Zhang Z, Miller W, Lipman DJ: **Gapped BLAST and PSI-BLAST: a new generation of protein database search programs.** *Nucleic Acids Res* 1997, **25:**3389-3402.
2. Hubbard TJ, Aken BL, Beal K, Ballester B, Caccamo M, Chen Y, Clarke L, Coates G, Cunningham F, Cutts T, et al: **Ensembl 2007.** *Nucleic Acids Res* 2007, **35:**D610-617.
3. Bratkovic T, Lunder M, Urleb U, Strukelj B: **Peptide inhibitors of MurD and MurE, essential enzymes of bacterial cell wall biosynthesis.** *J Basic Microbiol* 2008, **48:**202-206.
4. Kotnik M, Anderluh PS, Prezelj A: **Development of novel inhibitors targeting intracellular steps of peptidoglycan biosynthesis.** *Curr Pharm Des* 2007, **13:**2283-2309.
5. Helm JS, Hu Y, Chen L, Gross B, Walker S: **Identification of active-site inhibitors of MurG using a generalizable, high-throughput glycosyltransferase screen.** *J Am Chem Soc* 2003, **125:**11168-11169.
6. Zawadzke LE, Norcia M, Desbonnet CR, Wang H, Freeman-Cook K, Dougherty TJ: **Identification of an inhibitor of the MurC enzyme, which catalyzes an essential step in the peptidoglycan precursor synthesis pathway.** *Assay Drug Dev Technol* 2008, **6:**95-103.
7. Robertson D, Carroll P, Parish T: **Rapid recombination screening to test gene essentiality demonstrates that pyrH is essential in Mycobacterium tuberculosis.** *Tuberculosis (Edinb)* 2007, **87:**450-458.
8. Peukert S, Sun Y, Zhang R, Hurley B, Sabio M, Shen X, Gray C, Dzink-Fox J, Tao J, Cebula R, Wattanasin S: **Design and structure-activity relationships of potent and selective inhibitors of undecaprenyl pyrophosphate synthase (UPPS): tetramic, tetronic acids and dihydropyridin-2-ones.** *Bioorg Med Chem Lett* 2008, **18:**1840-1844.
9. Tong L, Harwood HJ, Jr.: **Acetyl-coenzyme A carboxylases: versatile targets for drug discovery.** *J Cell Biochem* 2006, **99:**1476-1488.
10. Tong L: **Acetyl-coenzyme A carboxylase: crucial metabolic enzyme and attractive target for drug discovery.** *Cell Mol Life Sci* 2005, **62:**1784-1803.
11. Almiron M, Martinez M, Sanjuan N, Ugalde RA: **Ferrochelatase is present in Brucella abortus and is critical for its intracellular survival and virulence.** *Infect Immun* 2001, **69:**6225-6230.
12. Capobianco JO, Darveau RP, Goldman RC, Lartey PA, Pernet AG: **Inhibition of exogenous 3-deoxy-D-manno-octulosonate incorporation into lipid A precursor of toluene-treated Salmonella typhimurium cells.** *J Bacteriol* 1987, **169:**4030-4035.
13. Ku MJ, Yoon HJ, Ahn HJ, Kim HW, Baek SH, Suh SW: **Crystallization and preliminary X-ray crystallographic studies of 3-deoxy-manno-octulosonate cytidylyltransferase from Haemophilus influenzae.** *Acta Crystallogr D Biol Crystallogr* 2003, **59:**180-182.
14. Jelakovic S, Schulz GE: **The structure of CMP:2-keto-3-deoxy-manno-octonic acid synthetase and of its complexes with substrates and substrate analogs.** *J Mol Biol* 2001, **312:**143-155.
15. Sinha I, Dick T: **Role for malonyl coenzyme A:acyl carrier protein transacylase (MCAT) in the growth-inhibitory effect of the calmodulin antagonist**

trifluoperazine in Mycobacterium bovis BCG. *J Antimicrob Chemother* 2004, **53:**1072-1075.
16. Sohn MJ, Zheng CJ, Kim WG: **Macrolactin S, a New Antibacterial Agent with FabG-inhibitory Activity from Bacillus sp. AT28.** *J Antibiot (Tokyo)* 2008, **61:**687-691.
17. Shi W, Bogdanov M, Dowhan W, Zusman DR: **The pss and psd genes are required for motility and chemotaxis in Escherichia coli.** *J Bacteriol* 1993, **175:**7711-7714.
18. Raetz CR, Foulds J: **Envelope composition and antibiotic hypersensitivity of Escherichia coli mutants defective in phosphatidylserine synthetase.** *J Biol Chem* 1977, **252:**5911-5915.
19. Belunis CJ, Clementz T, Carty SM, Raetz CR: **Inhibition of lipopolysaccharide biosynthesis and cell growth following inactivation of the kdtA gene in Escherichia coli.** *J Biol Chem* 1995, **270:**27646-27652.
20. Kumar P, Chhibber M, Surolia A: **How pantothenol intervenes in Coenzyme-A biosynthesis of Mycobacterium tuberculosis.** *Biochem Biophys Res Commun* 2007, **361:**903-909.
21. Kobayashi K, Ehrlich SD, Albertini A, Amati G, Andersen KK, Arnaud M, Asai K, Ashikaga S, Aymerich S, Bessieres P, et al: **Essential Bacillus subtilis genes.** *Proc Natl Acad Sci U S A* 2003, **100:**4678-4683.
22. Ji Y, Zhang B, Van SF, Horn, Warren P, Woodnutt G, Burnham MK, Rosenberg M: **Identification of critical staphylococcal genes using conditional phenotypes generated by antisense RNA.** *Science* 2001, **293:**2266-2269.
23. Chopra I: **Bacterial RNA polymerase: a promising target for the discovery of new antimicrobial agents.** *Curr Opin Investig Drugs* 2007, **8:**600-607.
24. Cornish RM, Roth JR, Poulter CD: **Lethal mutations in the isoprenoid pathway of Salmonella enterica.** *J Bacteriol* 2006, **188:**1444-1450.
25. Singh N, Cheve G, Avery MA, McCurdy CR: **Targeting the methyl erythritol phosphate (MEP) pathway for novel antimalarial, antibacterial and herbicidal drug discovery: inhibition of 1-deoxy-D-xylulose-5-phosphate reductoisomerase (DXR) enzyme.** *Curr Pharm Des* 2007, **13:**1161-1177.
26. Mao J, Eoh H, He R, Wang Y, Wan B, Franzblau SG, Crick DC, Kozikowski AP: **Structure-activity relationships of compounds targeting mycobacterium tuberculosis 1-deoxy-D-xylulose 5-phosphate synthase.** *Bioorg Med Chem Lett* 2008, **18:**5320-5323.
27. Srivastava A, Mukherjee P, Desai PV, Avery MA, Tekwani BL: **Structural analysis of farnesyl pyrophosphate synthase from parasitic protozoa, a potential chemotherapeutic target.** *Infect Disord Drug Targets* 2008, **8:**16-30.
28. Illarionova V, Kaiser J, Ostrozhenkova E, Bacher A, Fischer M, Eisenreich W, Rohdich F: **Nonmevalonate terpene biosynthesis enzymes as antiinfective drug targets: substrate synthesis and high-throughput screening methods.** *J Org Chem* 2006, **71:**8824-8834.
29. Sgraja T, Alphey MS, Ghilagaber S, Marquez R, Robertson MN, Hemmings JL, Lauw S, Rohdich F, Bacher A, Eisenreich W, et al: **Characterization of Aquifex aeolicus 4-diphosphocytidyl-2C-methyl-d-erythritol kinase - ligand recognition in a template for antimicrobial drug discovery.** *FEBS J* 2008, **275:**2779-2794.
30. Rohdich F, Bacher A, Eisenreich W: **Perspectives in anti-infective drug design. The late steps in the biosynthesis of the universal terpenoid precursors, isopentenyl diphosphate and dimethylallyl diphosphate.** *Bioorg Chem* 2004, **32:**292-308.
